# Mothers' anxiety during pregnancy is associated with asthma in their children

**DOI:** 10.1016/j.jaci.2009.01.042

**Published:** 2009-04

**Authors:** Hannah Cookson, Raquel Granell, Carol Joinson, Yoav Ben-Shlomo, A. John Henderson

**Affiliations:** a Department of Internal Medicine, Sir Charles Gairdner Hospital, Perth, Australia; b Department of Social Medicine, University of Bristol, Bristol, United Kingdom; c Department of Community-Based Medicine, University of Bristol, Bristol, United Kingdom

**Keywords:** Anxiety, pregnancy, prenatal programming, asthma, child, ALSPAC, Avon Longitudinal Study of Parents and Children, HPA, Hypothalamo-pituitary-adrenal, OR, Odds ratio

## Abstract

**Background:**

Maternal stress in early life has been associated with the development of asthma in children, although it is unclear whether there are any critical periods of exposure. The association of asthma with prenatal exposure to maternal stress has not been reported.

**Objective:**

We tested whether prenatal and postnatal anxiety and/or depression in pregnant women predicted the risk of their offspring developing asthma in childhood.

**Methods:**

The Avon Longitudinal Study of Parents and Children is a population-based birth cohort recruited during pregnancy. Data were available on maternal anxiety scores and asthma at age 7½ years in 5810 children. Anxiety was assessed at 18 and 32 weeks of gestation by using the validated Crown-Crisp Experiential Index. Asthma was defined at age 7½ years as doctor-diagnosed asthma with current symptoms or treatment in the previous 12 months. Multivariable logistic regression was used to determine the association of prenatal anxiety with asthma (odds ratio; 95% CI).

**Results:**

Independent of postnatal anxiety and adjusted for a number of likely confounders, there was a higher likelihood of asthma at age 7½ years (odds ratio, 1.64; 95% CI, 1.25-2.17) in children of mothers in the highest compared with lowest quartile of anxiety scores at 32 weeks of gestation, with evidence for a dose-response (*P* value for trend <0.001).

**Conclusions:**

Maternal anxiety symptoms as an indicator of stress during fetal life may program the development of asthma during childhood.

Asthma is an important health problem that accounts for an estimated annual healthcare budget in the United Kingdom of £817.4 million ($1600.9 million; €1043.2 million).^1^ A high proportion of asthma begins in childhood, but despite intense research interest in early-life risk factors, the causes of asthma in children remain largely unknown.

Psychological and emotional factors are recognized to trigger asthma exacerbations,^2^ and emotional stimuli have been shown to cause increased respiratory resistance in asthma.^3,4^ Surveys of adults and children have identified a high level of comorbidity between asthma and anxiety.^5,6^ This has been studied in the context of impact on asthma control,^7^ symptom perception, and quality of life,^8^ but there are few data on the temporal relationship of these disorders and the possibility that there exists a causal link between anxiety and asthma. It is conceivable that anxiety and asthma coexist because of shared environmental influences, such as early childhood adversity,^9^ or that anxiety could act through a variety of postulated pathways to alter the risk of asthma. Of particular interest is the possibility that parental psychological state could influence disease risk in their children, possibly through influences on corticotrophin-mediated stress responses. There is some evidence to support an association of anxiety disorders with dysregulation of corticotrophin releasing factor,^10^ and alteration of hypothalamo-pituitary-adrenal (HPA) axis regulation has been suggested as a plausible pathway for reported associations between parental stress and asthma or wheezing in children. Wright et al^11^ reported an increase in early childhood wheezing in association with caregiver stress in a high-risk cohort of infants, and Kozyrskyj et al^12^ have recently reported a positive association between maternal distress and asthma in children in an unselected, population-based cohort. However, there are no human studies to date that have investigated the association of prenatal maternal anxiety or depression with subsequent asthma in the offspring.

The existence of a programming effect of prenatal stress on fetal development is supported by reports that adult mammals exposed to prenatal stress have altered HPA^13^ and immune function^14^ after birth and may be predisposed to airway inflammation and hyperresponsiveness.^15^ Maternal anxiety during pregnancy is associated with raised blood cortisol levels in early pregnancy and appears to strengthen the correlation between maternal blood and amniotic fluid levels, possibly through an effect on placental function.^16^ Transplacental passage of cortisol could then have a programming effect on HPA axis development in the fetus, leading to alterations of stress responses after birth. The Avon Longitudinal Study of Parents and Children (ALSPAC) study was the first to show in human beings that maternal anxiety levels during pregnancy were associated with alterations of cortisol responses measured 10 years later in preadolescent children.^17^

We wished to test the hypothesis that exposure to maternal anxiety during pregnancy was associated with asthma in childhood. We were able to investigate this association in a large, population-based birth cohort in which women's reported anxiety and depression were evaluated prospectively during pregnancy and after the birth of their child and for which we had data on a large range of potential confounding factors.

## Methods

Subjects were members of the ALSPAC, a population-based birth cohort that recruited 14,541 pregnant women residents in Avon, United Kingdom, with expected dates of delivery from April 1, 1991, to December 31, 1992. There were 14,062 live born children, and 13,988 of these children were alive at age 1 year and subsequently followed up. The cohort has been followed since birth with annual questionnaires and, since age 7 years, with objective measures in annual research clinics. The study protocol has been published previously,^18^ and further details can be found at: http://www.bris.ac.uk/alspac. Ethical approval for all measures was obtained from the ALSPAC Ethics and Law Committee and from Local Research Ethics Committees.

### Psychological measures

Anxiety symptoms were assessed using the anxiety subscale of the Crown-Crisp Experiential Index, a validated self-rating inventory^19^ administered to women during pregnancy as part of a self-completion questionnaire at 18 weeks and 32 weeks of gestation. Because some women were recruited after 18 weeks of gestation, eligibility for the current study was based on the availability of anxiety symptom data at 18 and 32 weeks of gestation (n = 10,710 as shown in Table I). Maternal anxiety symptoms were also assessed 8 months after birth by using the same scale, and maternal depression symptoms were assessed at these times using the Edinburgh Postnatal Depression Scale.^20^ A life events inventory of 42 events was administered at 18 weeks of gestation to the mother, from which a life events score was derived (see Methods and Appendix E1 in the Online Repository at www.jacionline.org). Anxiety symptoms were measured in the mother's partner at 18 weeks of gestation by using the Crown-Crisp index, and a measure of the child's anxiety was obtained from the emotional symptoms subscale of the parent-reported Strengths and Difficulties Questionnaire at 47 months.^21^

### Outcomes

Current asthma at age 7½ years was defined as a report of doctor's diagnosis of asthma ever and either reported symptoms of wheeze or treatment for asthma in the previous 12 months on a questionnaire sent to mothers at 91 months after birth. Bronchial hyperresponsiveness was defined as a PD_20_ ≤1.2 mg methacholine measured at 8 years using the method of Yan et al.^22^ Atopy was defined as a positive response (≥2 mm weal) to any 1 of house dust mite *(Dermatophagoides pteronyssinus)*, grass, or cat allergen on skin prick test to a panel of as many as 14 allergens at age 7 years. We have previously reported that sensitization to 1 of these 3 allergens identifies more than 95% of those with any positive skin test result.^23^

### Confounders

Possible confounders or intermediaries of the relationship between maternal anxiety and asthma in the child that were considered were the mother's age, education, history of asthma or allergy, and smoking during pregnancy, all from maternal self-completion questionnaires. Maternal problems during pregnancy (hypertension, diabetes, and steroid prescription); multiple pregnancy; and the child's sex, gestation, and weight at delivery were ascertained from maternal health records.

### Analysis

Anxiety symptom scores were not normally distributed and were analyzed as a categorical scale based on quartiles. The primary outcome was current asthma at 7½ years, and secondary analyses were performed for asthma with bronchial hyperresponsiveness and atopic/nonatopic asthma, stratified according to the criteria detailed. Odds ratios (ORs) and 95% CIs were calculated by using logistic regression models with and without adjustment for confounders.

Our analytical strategy attempted to differentiate between 2 classes of life course models: a critical/sensitive period model versus an accumulation model.^24^ We examined the strength of association between reported anxiety symptoms and asthma in the prenatal and postnatal period with and without mutual adjustment. We further defined 4 mutually exclusive exposure groups on the basis of anxiety symptom scores above the median value for each period. These were (1) no anxiety symptoms in either period (baseline group), (2) prenatal only, (3) postnatal only, and (4) both prenatal and postnatal. We postulated *a priori* that a prenatal critical period model would show a similarly increased risk for 2 and 4, whereas an accumulation model would find that 4 had the strongest association. We also attempted to differentiate between state and trait anxiety symptoms by relating anxiety symptom scores to a life event score derived from the life events inventory at 18 weeks of gestation. Mothers in the lowest quartile were categorized to have anxiety trait and those in the upper quartiles to have reactive anxiety.

To test for confounding of maternal prenatal effect by shared environmental variables, analyses were adjusted for the mother's partner's self-reported anxiety symptom scores during pregnancy. This should show an increased risk through a common confounding factor—for example, adverse social circumstances—but could not influence intrauterine factors directly. Secondary analyses were also adjusted for children's scores on the emotional symptoms subscale of the Strengths and Difficulties Questionnaire as a possible intermediary between maternal anxiety and childhood asthma (see Methods in the Online Repository at www.jacionline.org for full details).

Finally, to consider the possibility of reporting bias, associations between maternal anxiety symptoms and reports of common childhood problems (wheeze, earache, and accidents) were investigated (see Methods in the Online Repository at www.jacionline.org).

All analyses were performed by using Stata v10 (StataCorp, College Station, Tex).

## Results

**Fig 1:**
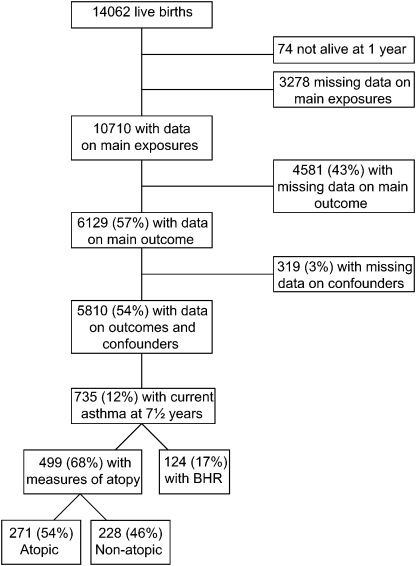
Number (%) of participants who completed both antenatal questionnaires on anxiety symptoms and had outcome data on child's asthma, bronchial responsiveness, and atopic status. *BHR*, bronchial hyperresponsiveness.

Both antenatal questionnaires were completed by 10,710 women. Complete data on maternal prenatal anxiety symptom scores at 18 and 32 weeks of gestation, asthma status of the offspring at 7½ years, and all confounder variables were available for 5810 (Fig 1). Mothers of children with incomplete data were likely to be younger, to have lower educational attainment, to smoke during pregnancy, and to report a personal history of asthma than those with complete data. They were also more likely to have higher anxiety symptom scores during and after pregnancy and higher depression symptom scores at 8 months after birth (Table I). Children with incomplete data were more likely to have lower birth weight, preterm delivery, and at least 1 sibling than those with complete data.

The associations between maternal prenatal anxiety symptoms and current asthma in children at 7½ years are shown in Table II. There was an increased prevalence of asthma associated with higher quartiles of anxiety symptom scores at both 18 and 32 weeks of gestation. There was only slight attenuation of the effect sizes with adjustment, and there appeared to be evidence of a dose-response gradient. For the more restrictive definition of asthma with bronchial hyperresponsiveness, the association with maternal anxiety symptoms at 32 weeks of gestation became stronger, whereas at 18 weeks it became more modest, and only the former showed a dose-response effect that was unlikely to be the result of chance (adjusted *P* value = .005). There were marginally greater effect sizes for anxiety symptoms not associated with life events (anxiety trait) compared with those that were (see this article's Table E1 in the Online Repository at www.jacionline.org), but there was little evidence of an interaction between maternal anxiety symptoms and life events on asthma risk (*P* = .4).

Analyses of asthma in children at 7½ years stratified by atopy showed similar trends for higher maternal anxiety quartiles to be associated with increased prevalence of both atopic and nonatopic asthma in their children at age 7½ years (see this article's Table E2 in the Online Repository at www.jacionline.org). There was stronger evidence for dose-response relationships for maternal anxiety symptoms reported at 32 weeks compared with 18 weeks, and effect sizes were greater for nonatopic compared with atopic asthma. *Post hoc* analysis (multinomial logistic regression) provided weak evidence for a stronger effect of anxiety symptoms at 18 weeks on nonatopic compared with atopic asthma (*P* = .05), but no evidence for a differential effect of anxiety symptoms at 32 weeks (*P* = .6). There was no strong evidence of associations between maternal anxiety symptoms and either allergy or bronchial hyperresponsiveness alone (see this article's Table E3 and Table E4 in the Online Repository at www.jacionline.org).

To investigate the effects of timing of exposure, we considered the associations between children's asthma and maternal anxiety symptom scores in the prenatal (32 weeks) and postnatal (8 months) periods with mutual adjustment (Table III). This showed that only prenatal anxiety symptoms were strongly associated with asthma in children after adjustment for postnatal anxiety symptoms and other confounders. Further analysis of timing of exposure showed that exposure in both prenatal and postnatal periods was more strongly associated with children's asthma (OR, 1.46; 95% CI, 1.20-1.78) than during either prenatal (OR, 1.30; 95% CI, 1.04-1.64) or postnatal (OR, 1.18; 95% CI 0.89-1.57) alone. However, further exploration of this relationship suggested that, rather than an accumulation effect, duration of anxiety symptoms was a marker of prenatal severity so that women reporting anxiety symptoms in both periods had higher anxiety scores at 32 weeks than those with anxiety symptoms at 32 weeks only, and hence their anxiety symptoms were more likely to persist (see this article's Table E5 in the Online Repository at www.jacionline.org).

Adjustment for the mother's partner's anxiety symptoms at 18 weeks of gestation as a test for confounding of prenatal maternal effects (see this article's Table E6 in the Online Repository at www.jacionline.org) showed no association between anxiety symptoms and children's asthma if only the partner reported anxiety symptoms (OR, 0.94; 95% CI, 0.73-1.20) compared with maternal anxiety symptoms alone (OR, 1.28; 95% CI, 1.00-1.63). However, if both partners reported anxiety symptoms (above median score) at 18 weeks, the effect was stronger (OR, 1.33; 95% CI, 1.04-1.68). This relationship was again confounded by severity of maternal anxiety symptoms because more anxious women were more likely to have anxious partners (see this article's Table E7 in the Online Repository at www.jacionline.org).

There was no evidence that the association between maternal anxiety symptoms and childhood asthma was mediated through children's internalizing symptoms because these did not attenuate the effect of maternal anxiety. The OR (95% CI) for the highest tertile of the emotional symptom subscale at 47 months (compared with the lowest tertile) was 1.21 (0.98-1.48; *P* [trend] = 0.1).

Finally, there was no evidence that women with higher reported anxiety symptom scores were more likely to present their child with wheeze to a doctor in either early (6-18 months) or later (69-81 months) childhood (highest vs lowest anxiety symptom score at 32 weeks gestation: OR [95% CI], 1.19 [0.92-1.54] and 1.13 [0.81-1.58], respectively; see further details in this article's Table E8 in the Online Repository at www.jacionline.org).

## Discussion

The results of our study support a positive association between maternal anxiety symptoms during pregnancy and subsequent asthma in the offspring during childhood. The strength of the associations, their consistency across different outcome measures, their robustness to adjustment for a wide range of confounding or mediating variables, and the evidence for a dose-response relationship raise the possibility that this association may be causal, although its mechanism remains speculative. Although others have reported links between maternal distress and asthma in children,^11,12^ we believe this is the first report of an association between antenatal exposure and subsequent asthma during childhood.

Wright et al^11^ first reported the association of parental stress, measured by using a 4-item scale of perceived stress by regular telephone interview, and wheezing in infancy. More recently, a study of maternal stress in early childhood in a large Canadian population-based cohort reported an association with asthma at age 7 years in their children.^12^ In this study, stress was defined as a combination of depression and anxiety using health care utilization data. The authors also reported a dose-response relationship between the duration of exposure and the prevalence of asthma and concluded that short-term exposure, limited to the first year after birth, was not related to the subsequent development of asthma. Consistent with our observations, there was a relationship between severity of anxiety/depression and duration of symptoms. We found a stronger relationship between maternal anxiety symptoms and asthma when anxiety symptoms had been present for longer, but this is likely to be explained by a severity effect so that mothers who reported continuing anxiety symptoms to the postnatal period had higher antenatal anxiety scores and severe prenatal anxiety is more likely to be associated with persistence of symptoms. When we adjusted postnatal associations between maternal anxiety symptoms and asthma in childhood for the prenatal measures, the effect was attenuated completely.

Another difference between our approach and previous studies was the use of anxiety symptoms alone rather than a combination of anxiety and depression as the primary exposure. This decision was reached on the basis of exploratory analyses of the relative contributions of reported anxiety and depression symptoms to the association with the primary outcome, in which we found that the inclusion of prenatal depression scores did not affect the association between anxiety symptoms and childhood asthma, and no independent association between childhood asthma and maternal reported depression symptoms was detected. In addition, others have reported associations between maternal anxiety in the prenatal period and childhood outcomes that would be consistent with an intrauterine effect of maternal stress on altered fetal physiology in this population.^25-27^ We believe our data to be consistent with an antenatal programming effect, although the precise mechanism of this has still to be explained. We carefully considered the possibility that this observation was confounded by adjusting for a large number of variables known to be associated with asthma and by using the anxiety symptom score of the mother's partner as a further test of confounding.^28^ If there was a true intrauterine effect, we would expect the observed association to be considerably stronger for the mother's than for the partner's anxiety, which is what our analyses indicated. One of the possible confounding variables considered by Kozyrskyj et al^12^ but not by us was the attendance of the child at day care. Although it might be supposed that mothers with higher reported levels of anxiety symptoms would be less likely to send their young children to preschool day care, the reported protective association of day care with subsequent asthma^29^ in children would be expected to attenuate associations between anxiety symptoms and asthma. However, there are discrepant findings that suggest day care attendance may increase the risk of asthma, at least in young children,^30^ and the associations between day care and asthma may be modified depending on family history of allergy.^31^ Therefore, we could not discount the possibility of spurious associations arising through this source of confounding. However, in *post hoc* analyses, we found no evidence of a relationship between maternal anxiety scores and reported day care attendance by their children (see this article's Table E9 in the Online Repository at www.jacionline.org).

Our study has several strengths, including recruitment during pregnancy, enabling the prospective ascertainment of maternal anxiety and depression symptoms, thus making the possibility of recall bias or reverse causation unlikely. The use of an objective measure of bronchial hyperresponsiveness also increased the association with anxiety at 32 weeks, presumably because of less measurement error in the outcome. However, in common with many large, population-based longitudinal surveys,^32^ there was a considerable loss of data, because of either incomplete ascertainment or loss to follow-up. Those with incomplete data were more likely to come from socially disadvantaged backgrounds and had evidence of higher maternal anxiety scores compared with the population with complete data. This introduces the possibility of selection bias, but one would have to postulate that the association between anxiety and asthma in subjects with missing data operated in the opposite direction (anxiety symptoms associated with reduced risk of asthma) to abolish the observed association, which seems unlikely. Furthermore, adjustment for a range of variables associated with social disadvantage produced very little attenuation of effect sizes in our primary analyses.

Another potential weakness of this study was the reliance on maternal self-report to define anxiety and depression symptoms. The scales used have been validated previously for self-completion, and these measures have been associated with a number of clinical and biochemical outcomes in a sample of our study population.^17^ Because recall bias is not possible, any measurement error is likely to be random and hence will make our observed associations conservative. Also, although we reported positive associations of maternal anxiety symptoms with asthma, the interpretation for these findings must be viewed with some caution. We were unable to ascertain the biological sources of anxiety in the women in our study and had no maternal or fetal biomarker data to confirm anxiety had a biological effect in this population. There is also the possibility of residual confounding through unmeasured social or lifestyle variables, although we found little evidence of attenuation with the markers that were considered in this study.

Wright et al^33^ identified associations between caregiver stress in early childhood and allergic responses in infants predisposed to asthma or atopy. It was suggested these were related to changes in neuroendocrine function, in particular through the HPA axis, and might influence immune development through alterations of cytokine responses.^34^ Another possible mechanism for a prenatal programming effect is through epigenetic regulation of the glucocorticoid receptor gene, which has been shown to occur in human beings.^35^ Emerging evidence suggests that environmental variables may alter methylation profiles of other genes of relevance to asthma,^36^ opening the possibility that maternal stress could operate through direct epigenetic effects, as well as indirect effects mediated through neuroendocrine dysregulation. Kozyrskyj et al^12^ reported effects of continued stress beyond the perinatal period on childhood asthma. It is possible to reconcile these results with our observations if postnatal behaviors modify the effects of prenatal anxiety,^37^ such as changing emotional attachments to the newborn infant, which could alter postpartum cortisol levels^38^; although in primate studies, high prenatal levels were associated with more attentive infant care behaviors. Early caregiving behavior can also modify neurobiological responses, including the response of the HPA axis to stressful stimuli.^39^ Thus, prenatal programming and postnatal behavioral modification could operate through similar pathways to influence fetal and child development.

Our data support an independent effect of prenatal exposure to maternal anxiety on the development of asthma during childhood, although we can only speculate on the mechanisms of this association, and we hope in future studies to have biomarker data on cortisol responses in children to explore this further. We did not find evidence to suggest a stronger association in subjects reporting atopic asthma, suggesting that the association between maternal anxiety and asthma is not mediated directly through influences on allergic sensitization. There has been a continuing interest in pregnancy as a critical period in the development of asthma in children, and emerging evidence supports the possibility of programming effects of maternal stress in pregnancy on several childhood outcomes. The development of relatively straightforward interventions to reduce maternal stress in pregnancy makes it possible to test these hypothetical relationships in a controlled trial.^40^ Although it is too early to advocate specific interventions aimed at primary prevention of asthma, we would suggest that asthma and allergic outcomes are included if intervention studies to reduce anxiety and distress during pregnancy are considered.

**Clinical implications**

These results indicate a biologically plausible risk factor for asthma that is amenable to prenatal intervention.

## Figures and Tables

**Table:** Associations of maternal anxiety symptoms at 18 weeks of gestation (stratified by reactive and nonreactive to life events) with children's asthma at 7½ years

	OR (95% CI) for current asthma at 7½ y		
	N asthma/no asthma (% with asthma)	Crude	Adjusted∗
Maternal reactive anxiety at 18 wk			
1st quartile (0-2)	99/781 (11.3)	1 (reference)	1 (reference)
2nd quartile (3-4)	100/661 (13.1)	1.19 (0.89-1.61)	1.13 (0.84-1.53)
3rd quartile (5-7)	131/845 (13.4)	1.22 (0.93-1.62)	1.20 (0.90-1.59)
4th quartile (8-16)	127/660 (16.1)	1.52 (1.14-2.01)	1.38 (1.03-1.85)
*P* (trend)		.005	.029
Maternal anxiety trait at 18 wk			
1st quartile (0-2)	99/948 (9.5)	1 (reference)	1 (reference)
2nd quartile (3-4)	73/512 (12.5)	1.37 (0.99-1.88)	1.33 (0.96-1.85)
3rd quartile (5-7)	68/444 (13.3)	1.47 (1.06-2.04)	1.46 (1.04-2.05)
4th quartile (8-16)	36/207 (14.8)	1.67 (1.11-2.51)	1.71 (1.12-2.62)
*P* (trend)		.004	.004

∗ Adjusted for sex, preterm delivery, multiple birth, number of siblings, maternal age, maternal education, maternal history of asthma and allergy, prenatal tobacco smoke exposure, and problems during pregnancy (diabetes, hypertension, steroid intake).

**Table:** Associations between maternal anxiety symptoms at 18 and 32 weeks of gestation and asthma with atopy in children age 7½ years

	OR (95% CI) for atopic asthma at 7½ y			OR (95% CI) for nonatopic asthma at 7½ y		
	N asthma/no asthma (% with asthma)	Crude	Adjusted∗	N asthma/no asthma (% with asthma)	Crude	Adjusted∗
Maternal anxiety at 18 wk						
1st quartile (0-2)	80/1734 (4.4%)	1 (reference)	1 (reference)	66/1734 (3.7%)	1 (reference)	1 (reference)
2nd quartile (3-4)	72/1176 (5.8%)	1.33 (0.96-1.84)	1.27 (0.91-1.77)	42/1176 (3.4%)	0.94 (0.63-1.39)	0.92 (0.62-1.37)
3rd quartile (5-7)	68/1295 (5%)	1.14 (0.82-1.59)	1.11 (0.79-1.56)	58/1295 (4.3%)	1.18 (0.82-1.69)	1.17 (0.81-1.68)
4th quartile 8-16)	51/870 (5.5%)	1.27 (0.89-1.82)	1.20 (0.83-1.74)	62/870 (6.7%)	1.87 (1.31-2.67)	1.78 (1.24-2.57)
*P* (trend)		.3	.4		<.001	.002
Maternal anxiety at 32 wk						
1st quartile (0-2)	70/1582 (4.2%)	1 (reference)	1 (reference)	47/1582 (2.9%)	1 (reference)	1 (reference)
2nd quartile (3-4)	62/1232 (4.8%)	1.14 (0.80-1.61)	1.08 (0.76-1.54)	59/1232 (4.6%)	1.61 (1.09-2.38)	1.57 (1.06-2.33)
3rd quartile (5-7)	76/1308 (5.5%)	1.31 (0.94-1.83)	1.27 (0.90-1.77)	68/1308 (4.9%)	1.75 (1.20-2.56)	1.72 (1.17-2.51)
4th quartile 8-16)	63/953 (6.2%)	1.49 (1.05-2.12)	1.43 (1.00-2.05)	54/953 (5.4%)	1.91 (1.28-2.84)	1.80 (1.20-2.70)
*P* (trend)		.02	.03		.001	.003

∗ Adjusted for sex, preterm delivery, multiple birth, number of siblings, maternal age, maternal education, maternal history of asthma and allergy, prenatal tobacco smoke exposure, and problems during pregnancy (diabetes, hypertension, steroid intake)

**Table:** Association between maternal anxiety symptoms and allergy∗ in children

	OR (95% CI) for allergy at 7½ y		
	N allergy/no allergy (% with allergy)	Crude	Adjusted†
Maternal anxiety symptoms at 18 wk			
1st quartile (0-2)	384/1387 (21.7)	1 (reference)	1 (reference)
2nd quartile (3-4)	255/1058 (19.4)	0.87 (0.73-1.04)	0.86 (0.72-1.03)
3rd quartile (5-7)	310/1121 (21.7)	1.00 (0.84-1.18)	1.00 (0.84-1.19)
4th quartile (8-16)	215/865 (19.9)	0.90 (0.74-1.08)	0.95 (0.78-1.15)
*P* (trend)		.50	.87
Maternal anxiety symptoms at 32 wk			
1st quartile (0-2)	372/1277 (22.6)	1 (reference)	1 (reference)
2nd quartile (3-4)	282/1138 (19.9)	0.85 (0.71-1.01)	0.88 (0.74-1.05)
3rd quartile (5-7)	292/1237 (19.1)	0.81 (0.68-0.96)	0.83 (0.70-0.99)
4th quartile (8-16)	260/911 (21.6)	0.98 (0.82- 1.17)	1.06 (0.88-1.27)
*P* (trend)		.47	.99

∗ Allergy defined as positive skin prick test result to 1 of cat/grass/house dust mite.

† Adjusted for sex, preterm delivery, multiple birth, number of siblings, maternal age, maternal education, maternal history of asthma and allergy, prenatal tobacco smoke exposure, and problems during pregnancy (diabetes, hypertension, steroid intake).

**Table:** Association between maternal anxiety symptoms and bronchial hyperresponsiveness∗ in children

	OR (95% CI) for bronchial hyperresponsiveness (BHR) at 8½ y		
	N BHR/no BHR (% with BHR)	Crude	Adjusted†
Maternal anxiety symptoms at 18 wk			
1st quartile (0-2)	197/1051 (15.8)	1 (reference)	1 (reference)
2nd quartile (3-4)	139/773 (15.2)	0.96 (0.76-1.21)	0.95 (0.75-1.20)
3rd quartile (5-7)	146/807 (15.3)	0.97 (0.76-1.22)	0.96 (0.76-1.21)
4th quartile (8-16)	120/555 (17.8)	1.15 (0.90-1.48)	1.15 (0.89-1.48)
*P* (trend)		.39	.42
Maternal anxiety symptoms at 32 wk			
1st quartile (0-2)	190/1004 (15.9)	1 (reference)	1 (reference)
2nd quartile (3-4)	143/804 (15.1)	0.94 (0.74-1.19)	0.95 (0.75-1.21)
3rd quartile (5-7)	158/871 (15.4)	0.96 (0.76-1.21)	0.97 (0.77-1.22)
4th quartile (8-16)	130/618 (17.4)	1.11 (0.87-1.42)	1.14 (0.89-1.46)
*P* (trend)		.50	.41

∗ Bronchial hyperresponsiveness defined as PD_20_ <1.2 mg methacholine.

† Adjusted for sex, preterm delivery, multiple birth, number of siblings, maternal age, maternal education, maternal history of asthma and allergy, prenatal tobacco smoke exposure, and problems during pregnancy (diabetes, hypertension, steroid intake).

**Table:** Comparison of proportions in upper quartiles of maternal anxiety symptoms at 32 weeks for women with reported prenatal anxiety∗ alone or with prenatal and postnatal anxiety∗

	Maternal anxiety at 32 wk	
	3rd quartile (5-7)	4th quartile (8-16)
Accumulated maternal anxiety		
32 wk pregnancy only	720 (70%)	302 (30%)
32 wk pregnancy and 8 mo	724 (49%)	762 (51%)

∗ Greater than median value for each period. N (%) in table refers to number of women (%) in the upper two quartiles of anxiety symptoms scores at 32 weeks of gestation.

**Table:** Association of mothers' and partners' anxiety symptom scores at 18 weeks of gestation and current asthma in children at 7½ years

	OR (95% CI) for current asthma at 7½ y		
	N asthma/no asthma (% with asthma)	Crude	Adjusted
Combined parental anxiety at 18 weeks gestation∗			
Neither parent anxious	173/1384 (11.1%)	1 (reference)	1 (reference)
Only partner anxious	121/994 (10.9%)	0.97 (0.76-1.25)	0.94 (0.73-1.20)
Only mother anxious	137/836 (14.1%)	1.31 (1.03-1.67)	1.28 (1.00-1.63)
Both parents anxious	154/889 (14.8%)	1.39 (1.10-1.75)	1.33 (1.04-1.68)
*P* (trend)		.001	.005

∗ Anxiety defined as present if score above median.

**Table:** Distribution of mothers' and their partners' anxiety symptom categories at 18 weeks of gestation

	Paternal anxiety at 18 wk **(%)**			
	1st quartile (0-1)	2nd quartile (2)	3rd quartile (3-4)	4th quartile (5-12)
Maternal anxiety at 18 wk				
1st quartile (0-2)	44.0	16.8	19.5	19.8
2nd quartile (3-4)	38.4	16.3	21.0	24.2
3rd quartile (5-7)	35.1	15.6	22.6	26.7
4th quartile (8-16)	29.8	14.9	24.7	30.6

**Table:** Doctor consultations for common childhood problems in early and late childhood according to maternal prenatal anxiety symptom scores

	OR (95% CI) for wheezing			OR (95% CI) for earache			OR (95% CI) for accident		
	N (%)	Crude	Adjusted for sex	N (%)	Crude	Adjusted for sex	N (%)	Crude	Adjusted for sex
Maternal anxiety at 18 wk	Symptoms between 6 and 18 mo								
1st quartile (0-2)	758/912 (83.1)	1.0 (reference)	1.0 (ref)	924/988 (93.5)	1.0 (reference)	1.0 (reference)	356/567 (62.8)	1.0 (reference)	1.0 (reference)
2nd quartile (3-4)	611/732 (83.5)	1.03 (0.79-1.33)	1.03 (0.79-1.34)	733/807 (90.8)	0.69 (0.48-0.97)	0.69 (0.48-0.97)	294/456 (64.5)	1.08 (0.83-1.39)	1.08 (0.83-1.40)
3rd quartile (5-7)	770/914 (84.2)	1.09 (0.85-1.39)	1.09 (0.85-1.40)	903/978 (92.3)	0.83 (0.59-1.18)	0.83 (0.59-1.18)	386/584 (66.1)	1.16 (0.91-1.47)	1.16 (0.91-1.47)
4th quartile (8-16)	735/848 (86.7)	1.32 (1.02-1.72)	1.33 (1.02-1.73)	773/868 (89.1)	0.56 (0.40-0.78)	0.56 (0.40-0.78)	354/490 (72.2)	1.54 (1.19-2.0)	1.55 (1.19-2.01)
*P* trend		.04	.04		.004	.004		.002	.001
Maternal anxiety at 18 wk	Symptoms between 69 and 81 mo								
1st quartile (0-2)	243/351 (69.2)	1.0 (reference)	1.0 (reference)	739/1016 (72.7)	1.0 (reference)	1.0 (reference)	318/444 (71.6)	1.0 (reference)	1.0 (reference)
2nd quartile (3-4)	205/314 (65.3)	0.84 (0.60-1.16)	0.83 (0.60-1.15)	567/777 (73.0)	1.01 (0.82-1.25)	1.01 (0.82-1.25)	208/317 (65.6)	0.76 (0.55-1.03)	0.75 (0.55-1.03)
3rd quartile (5-7)	246/383 (64.2)	0.80 (0.59-1.09)	0.78 (0.57-1.07)	696/950 (73.3)	1.03 (0.84-1.25)	1.03 (0.82-1.25)	271/416 (65.1)	0.74 (0.55-0.99)	0.74 (0.55-0.99)
4th quartile (8-16)	213/310 (68.7)	0.98 (0.70-1.36)	0.97 (0.70-1.35)	527/689 (76.5)	1.22 (0.97-1.53)	1.22 (0.97-1.53)	196/313 (62.6)	0.66 (0.49-0.90)	0.66 (0.49-0.90)
*P* trend		.74	.69		.12	.12		.009	.009
Maternal anxiety at 32 wk	Symptoms between 6 and 18 mo								
1st quartile (0-2)	658/793 (83.0)	1.0 (reference)	1.0 (reference)	858/927 (92.6)	1.0 (reference)	1.0 (reference)	325/526 (61.8)	1.0 (reference)	1.0 (reference)
2nd quartile (3-4)	624/749 (83.3)	1.02 (0.78-1.34)	1.03 (0.79-1.34)	762/836 (91.1)	0.83 (0.59-1.17)	0.83 (0.59-1.16)	333/497 (67.0)	1.26 (0.97-1.62)	1.26 (0.97-1.62)
3rd quartile (5-7)	812/971 (83.6)	1.05 (0.81-1.35)	1.05 (0.82-1.36)	931/1021 91.2)	0.83 (0.60-1.15)	0.83 (0.60-1.15)	376/579 (64.9)	1.15 (0.90-1.46)	1.15 (0.90-1.46)
4th quartile (8-16)	813/954 (85.2)	1.18 (0.91-1.53)	1.19 (0.92-1.54)	864/960 (90)	0.72 (0.52-1.0)	0.72 (0.52-1.0)	394/556 (70.8)	1.50 (1.17-1.94)	1.50 (1.17-1.94)
*P* trend		.20	.19		.06	.06		.006	.006
Maternal anxiety at 32 wk	Symptoms between 69 and 81 mo								
1st quartile (0-2)	213/313 (68.1)	1.0 (reference)	1.0 (reference)	681/939 (72.5)	1.0 (reference)	1.0 (reference)	286/405 (70.6)	1.0 (reference)	1.0 (reference)
2nd quartile (3-4)	209/322 (64.9)	0.87 (0.62-1.21)	0.86 (0.61-1.19)	607/824 (73.7)	1.06 (0.86-1.31)	1.06 (0.86-1.31)	247/366 (67.5)	0.86 (0.64-1.17)	0.86 (0.63-1.17)
3rd quartile (5-7)	260/400 (65.0)	0.87 (0.64-1.19)	0.85 (0.62-1.16)	727/993 (73.2)	1.04 (0.85-1.27)	1.04 (0.85-1.27)	275/409 (67.2)	0.85 (0.63-1.15)	0.85 (0.63-1.15)
4th quartile (8-16)	249/351 (70.9)	1.15 (0.82-1.60)	1.13 (0.81-1.58)	583/762 (76.5)	1.23 (0.99-1.54)	1.23 (0.99-1.54)	212/355 (59.7)	0.62 (0.46-0.83)	0.62 (0.46-0.83)
*P* trend		.43	.48		.10	.10		.003	.003

**Table:** Distribution of maternal prenatal (32 weeks) and postnatal (8 months) anxiety symptom scores and day care attendance of preschool child

	Day care attendance				Day care attendance		
	Yes	No	Total		Yes	No	Total
	N (%)	N (%)	N	Maternal anxiety at 8 mo	N (%)	N (%)	N
Maternal anxiety at 32 wk							
1st quartile	180 (6.4)	2,647 (93.6)	2,827	1st quartile	192 (5.8)	3,114 (94.2)	3,306
2nd quartile	138 (5.9)	2,202 (94.1)	2,340	2nd quartile	187 (6.5)	2,699 (93.5)	2,886
3rd quartile	168 (6.4)	2,479 (93.6)	2,647	3rd quartile	100 (5.9)	1,602 (94.1)	1,702
4th quartile	115 (5.4)	2,033 (94.7)	2,148	4th quartile	144 (6.4)	2,114 (93.6)	2,258
			9,962				10,152

**Table I:** Comparison of population with complete data with those with incomplete data or lost to follow-up

	Original cohort N = 10,710	Study population N = 5,810∗	Incomplete N = 4,900	*P* value‡
Prenatal measures				
Main exposures				
Maternal anxiety at 18 wk				
1st quartile (0-2)	3,265 (30.5)	1,932 (33.3)	1,333 (27.2)	
2nd quartile (3-4)	2,411 (22.5)	1,350 (23.2)	1,061 (21.7)	<.001
3rd quartile (5-7)	2,814 (26.3)	1,495 (25.7)	1,319 (26.9)	
4th quartile (8-16)	2,220 (20.7)	1,033 (17.8)	1,187 (24.2)	
Maternal anxiety at 32 wk				
1st quartile (0-2)	2,981 (27.8)	1,750 (30.1)	1,231 (25.1)	
2nd quartile (3-4)	2,481 (23.2)	1,417 (24.4)	1,064 (21.7)	<.001
3rd quartile (5-7)	2,836 (26.5)	1,512 (26.0)	1,324 (27.0)	
4th quartile (8-16)	2,412 (22.5)	1,131 (19.5)	1,281 (26.1)	
Confounders				
Maternal age (mean [SD])	28.4 (4.8)	29.2 (4.5)	27.4 (5.0)	<.001
Low birth weight <2.5 kg	498/10,577 (4.7)	247 (4.3)	251/4,767 (5.3)	.01
Sex (males)	5,539/10,709 (51.7)	2,985 (51.4)	2,554/4,899 (52.1)	.4
Preterm <37 wk	599 (5.6)	296 (5.1)	303 (6.2)	.02
Multiple birth	283 (2.6)	136 (2.3)	147 (3.0)	.03
≥1 Sibling	5,862/10,603 (55.3)	3,127 (53.8)	2,735/4,793 (57.1)	.001
Maternal low education†	6,822/10,657 (64.0)	3,343 (57.5)	3,479/4,847 (71.8)	<.001
Maternal asthma	1,166/10,265 (11.4)	610 (10.5)	556/4,455 (12.5)	.002
Maternal allergy	4,411/10,224 (43.1)	2,551 (43.9)	1,860/4,414 (42.1)	.07
Maternal smoking in pregnancy	3,459 (32.3)	1,505 (25.9)	1,954 (39.9)	<.001
Diabetes during pregnancy	52 (0.5)	30 (0.5)	22 (0.4)	.6
Hypertension during pregnancy	1,031 (9.6)	529 (9.1)	502 (10.2)	.05
Steroids during pregnancy	60 (0.6)	29 (0.5)	31 (0.6)	.4
Other exposures				
Partner's anxiety at 18 wk	N = 8,139	N = 4,688	N = 3,451	
1st quartile (0-1)	3,089 (38.0)	1,778 (37.9)	1,311 (38.0)	
2nd quartile (2)	1,341 (16.5)	752 (16.0)	589 (17.1)	.6
3rd quartile (3-4)	1,744 (21.4)	1,011 (21.6)	733 (21.2)	
4th quartile (5-16)	1,965 (24.1)	1,147 (24.5)	818 (23.7)	
Postnatal measures				
Main outcomes				
Current asthma at 7½ y	781/6,129 (12.7)	735/5,810 (12.7)	46/319 (14.4)	.4
Current asthma with bronchial hyperresponsiveness	130/5,478 (2.4)	124/5,199 (2.4)	6/279 (2.2)	.8
Atopic asthma	285/5,596 (5.1)	271/5,303 (5.1)	14/293 (4.9)	.9
Nonatopic asthma	248/5,633 (4.4)	228/5,346 (4.3)	20/287 (6.8)	.04
Other exposures				
Maternal anxiety at 8 mo	N = 9,412	N = 5,543	N = 3,869	
1st quartile (0-1)	3,087 (32.8)	1,864 (33.6)	1,223 (31.6)	
2nd quartile (2-3)	2,689 (28.6)	1,615 (29.1)	1,074 (27.8)	.001
3rd quartile (4-5)	1,590 (16.9)	933 (16.8)	657 (17.0)	
4th quartile (6-16)	2,046 (21.7)	1,131 (20.4)	915 (23.6)	
Maternal depression at 8 mo	N = 9,421	N = 5552	N = 3,869	
1st quartile (0-2)	3,147 (33.4)	1,918 (34.5)	1,229 (31.8)	
2nd quartile (3-4)	1,780 (18.9)	1,083 (19.5)	697 (18.0)	<.001
3rd quartile (5-8)	2,504 (26.6)	1,472 (26.5)	1,032 (26.7)	
4th quartile (9-29)	1,990 (21.1)	1,079 (19.4)	911 (23.5)	
Children's internalizing symptoms	N = 8,046	N = 5,312	N = 2,734	
1st tertile (0-1)	4,940 (61.4)	3,284 (61.8)	1,656 (60.6)	
2nd tertile (2)	1,457 (18.1)	959 (18.1)	498 (18.2)	.5
3rd tertile (3-10)	1,649 (20.5)	1,069 (20.1)	580 (21.2)	

n/N (%) corresponds to number of positive cases, total, and percentage (N as specified in column heading when not indicated).

∗ Children with data on maternal anxiety symptoms at both 18 and 32 weeks of gestation, current asthma at 7½ years, and all confounders.

† Full time education up to and including General Certificate of Education (equivalent to school leaving certificate at age 16 y).

‡ *P* value for *t* test for comparison of means or χ^2^ test for comparison of proportions.

**Table II:** Association between maternal anxiety symptoms at 18 weeks and 32 weeks of gestation and asthma in children age 7½ years

	OR (95% CI) for asthma at 7½ y			OR (95% CI) for asthma with BHR		
	N asthma/no asthma (% with asthma)	Crude	Adjusted∗	N asthma/no asthma (% with asthma)	Crude	Adjusted∗
Maternal anxiety at 18 wk						
1st quartile (0-2)	198/1734 (10.2%)	1 (reference)	1 (reference)	38/1734 (2.1%)	1 (reference)	1 (reference)
2nd quartile (3-4)	174/1176 (12.9%)	1.30 (1.04-1.61)	1.24 (1.00-1.55)	33/1176 (2.7%)	1.28 (0.80-2.05)	1.22 (0.76-1.97)
3rd quartile (5-7)	200/1295 (13.4%)	1.35 (1.10-1.67)	1.32 (1.07-1.63)	29/1295 (2.2%)	1.02 (0.63-1.67)	0.99 (0.60-1.62)
4th quartile (8-16)	163/870 (15.8%)	1.64 (1.31-2.05)	1.53 (1.22-1.93)	24/870 (2.7%)	1.26 (0.75-2.11)	1.24 (0.73-2.11)
*P* (trend)		<.001	<.001		.6	.6
Maternal anxiety at 32 wk						
1st quartile (0-2)	168/1582 (9.6%)	1 (reference)	1 (reference)	27/1582 (1.7%)	1 (reference)	1 (reference)
2nd quartile (3-4)	185/1232 (13.1%)	1.41 (1.13-1.77)	1.36 (1.09-1.71)	29/1232 (2.3%)	1.38 (0.81-2.34)	1.35 (0.79-2.30)
3rd quartile (5-7)	204/1308 (13.5%)	1.47 (1.18-1.82)	1.42 (1.14-1.77)	35/1308 (2.6%)	1.57 (0.94-2.60)	1.55 (0.93-2.58)
4th quartile 8-16)	178/953 (15.7%)	1.76 (1.40-2.20)	1.65 (1.30-2.08)	33/953 (3.3%)	2.03 (1.21-3.40)	2.09 (1.24-3.53)
*P* (trend)		<.001	<.001		.006	.005

∗ Adjusted for sex, preterm delivery, multiple birth, number of siblings, maternal age, maternal education, maternal history of asthma and allergy, prenatal tobacco smoke exposure, and problems during pregnancy (diabetes, hypertension, steroid intake).

**Table III:** Relationship between maternal anxiety symptoms reported before (32 weeks of gestation) and after birth (8 months) and asthma in children age 7½ years

	OR (95% CI) for current asthma at 7½ y			
	N asthma/no asthma (% with asthma)	Crude	Adjusted∗	Adjusted for confounders∗ and for prenatal or postnatal anxiety
Maternal anxiety at 32 wk gestation				
1st quartile (0-2)	156/1529 (9.3%)	1 (reference)	1 (reference)	1 (reference)
2nd quartile (3-4)	166/1184 (12.3%)	1.37 (1.09-1.73)	1.33 (1.05-1.68)	1.31 (1.03-1.67)
3rd quartile (5-7)	193/1251 (13.4%)	1.51 (1.21-1.89)	1.46 (1.16-1.83)	1.42 (1.11-1.81)
4th quartile (8-16)	164/900 (15.4%)	1.79 (1.41-2.26)	1.68 (1.32-2.13)	1.64 (1.25-2.17)
*P* (trend)		<.001	<.001	<.001
Maternal anxiety at 8 mo after birth				
1st quartile (0-2)	196/1668 (10.5%)	1 (reference)	1 (reference)	1 (reference)
2nd quartile (3-4)	188/1427 (11.6%)	1.12 (0.91-1.39)	1.09 (0.88-1.35)	0.99 (0.80-1.24)
3rd quartile (5-7)	138/795 (14.8%)	1.48 (1.17-1.87)	1.40 (1.10-1.77)	1.18 (0.92-1.53)
4th quartile (8-16)	157/974 (13.9%)	1.37 (1.10-1.72)	1.26 (1.00-1.59)	0.99 (0.76-1.29)
*P* (trend)		<.001	.01	.7

∗ Adjusted for sex, preterm delivery, multiple birth, number of siblings, maternal age, maternal education, maternal history of asthma and allergy, prenatal tobacco smoke exposure, and problems during pregnancy (diabetes, hypertension, steroid intake).

## Methods

To explore the possible differences between anxiety trait and reactive anxiety to recent life events, we used a life events inventory administered to pregnant women at 18 weeks of gestation. This considered the occurrence of 42 events (Appendix E1) since pregnancy onset, modeled on a previously reported life events inventory.^E1^ A weighted life events score was derived by summing the self-reported impact of each event as follows: affected a lot = 4; fairly affected = 3; mildly affected = 2; no effect at all = 1; and if the event did not happen, it was coded = 0. Women in the lowest quartile of life events score (score = 0-2) were categorized as anxiety trait (nonreactive), and those in the upper 3 quartiles were categorized as having reactive anxiety symptoms. Associations with children's asthma for each of these categories and the interaction between anxiety symptom scores and life events on childhood asthma were tested.

To consider the timing of exposure to maternal anxiety, we created 4 mutually exclusive groups based on prenatal (32 weeks of gestation) and postnatal (8 months after birth) anxiety scores. Women whose anxiety score on the Crown-Crisp Experiential Index was greater than the group median score for each period were classed as anxiety present and those below the median as anxiety absent for each period. The relationship between each anxiety category and asthma at age 7½ years: prenatal only (32 weeks and not 8 months), postnatal only (8 months and not 32 weeks), and both (32 weeks and 8 months) were compared with those without anxiety at either period using logistic regression analysis.

To consider the possibility that the relationship between maternal anxiety reported in the antenatal period was confounded by variables other than those that were used in the adjusted multivariable analysis, we considered the mother's partner's anxiety score at 18 weeks of gestation as an adjustment variable. If there was a true intrauterine effect, we would expect the association with asthma to be stronger for maternal anxiety than for paternal anxiety.^E2^ Reported anxiety scores for mother and partner at 18 weeks of gestation were dichotomized above and below the median value for each group of subjects. Four mutually exclusive groups were created: neither mother nor partner anxious, mother only anxious, partner only anxious, and both mother and partner anxious. The association between each anxiety category and asthma at age 7½ years was compared with the neither category by logistic regression.

To determine whether the relationship between maternal anxiety scores and asthma at age 7½ years was likely to be mediated through anxious mothers having more anxious children, we used the emotional difficulties subscale of the Strengths and Difficulties Questionnaire^E3^ as a measure of internalizing symptoms. The questionnaire was posted as part of a larger self-completion questionnaire to mothers when their children were approximately 4 years (47 months) old. The responses were divided into 3 quantiles, and the relationship between these and subsequent asthma at age 7½ years was analyzed by using logistic regression.

To test for reporting bias, associations between maternal anxiety and 2 common childhood problems (earache and accidents), in addition to reported wheeze, obtained from self-completion questionnaires sent to the mother at 18 months (covering the period 6-18 months) and 81 months (covering the period 69-81 months). For each problem the mother was asked about its occurrence (yes/no) and if yes, whether she took the child to see a doctor. The occurrence of each problem during each of the 2 periods was compared across quartiles of maternal anxiety scores during early (18 weeks) and late (32 weeks) gestation of pregnancy. Logistic regression was used to determine whether mothers with greater anxiety scores were more likely to present their child to a doctor with each problem by comparing each anxiety quartile with the lowest quartile for that period.

All analyses were performed by using Stata v10 (StataCorp, College Station, Texas).

## Results

Table E1 shows the associations of children's asthma at age 7½ years with maternal anxiety at 18 weeks of gestation stratified by anxiety trait and reactive anxiety. Nonreactive anxiety symptoms (anxiety trait) was more strongly associated with children's asthma for all levels of anxiety symptom scores, but there was weak evidence of an interaction between anxiety symptoms and life events on children's asthma risk (*P* = .4).

Analysis of timing of exposure showed that anxiety in both prenatal and postnatal periods was more strongly associated with asthma (OR, 1.46; 95% CI, 1.20-1.78) than during either prenatal (OR, 1.30; 95% CI, 1.04-1.64) or postnatal (OR, 1.18; 95% CI, 0.89-1.57) alone. However, further exploration of this relationship suggested that it was confounded by severity; women reporting anxiety in both periods had higher anxiety scores at 32 weeks than those with anxiety at 32 weeks only (Table E5).

Adjustment of associations of maternal prenatal anxiety with asthma by the mother's partner's reported anxiety at 18 weeks of gestation (Table E6) showed no association between anxiety and asthma if only the partner reported anxiety (OR, 0.94; 95% CI, 0.73-1.20) compared with maternal anxiety alone at 18 weeks of gestation (OR, 1.28; 95% CI, 1.00-1.63). However, if both partners reported anxiety (above median score) at 18 weeks, the effect was marginally stronger (OR, 1.33; 95% CI, 1.04-1.68). This relationship was confounded by severity of maternal anxiety, with more anxious women being more likely to have anxious partners (Table E7).

There was no evidence that the association between maternal anxiety and childhood asthma was mediated through children's internalizing symptoms. The OR (95% CI) for the highest tertile of the emotional symptom subscale of the Strengths and Difficulties Questionnaire in children age 47 months (compared with lowest tertile) was 1.21 (0.98-1.48; *P* [trend] = 0.1).

Table E8 shows the proportions of children reported to have been taken to the doctor for wheeze, earache, or accidents in the periods 6 to 18 months and 69 to 81 months. There was no consistent pattern of increased numbers of children with each problem being reported in the higher quartiles of reported maternal anxiety scores at either 18 or 32 weeks of gestation. There was a tendency for mothers with higher anxiety scores to present younger children to doctors for accidents, but this appeared to reverse for the presentation of older children (69-81 months). There was also increased presentation of young children with wheeze in the highest quartile of maternal anxiety at 18 weeks but not at 32 weeks. For earache, it appeared that more anxious mothers were less likely to take young children to the doctor.

Table E9 shows an absence of association between reported maternal anxiety during pregnancy (32 weeks) or after birth (8 months) with whether their child attended day care.

## Appendix E1 Items in the life event inventory administered to pregnant women at 18 weeks of gestation

1. Your partner died
2. One of your children died
3. A friend or relative died
4. One of your children was ill
5. Your partner was ill
6. A friend or relative was ill
7. You were admitted to hospital
8. You were admitted to hospital
9. You were divorced
10. You found that your partner didn't want your child
11. You were very ill
12. Your partner lost his job
13. Your partner had problems at work
14. You had problems at work
15. You lost your job
16. Your partner went away
17. Your partner was in trouble with the law
18. You and your partner separated
19. Your income was reduced
20. You argued with your partner
21. You had arguments with your family or friends
22. You moved house
23. Your partner hurt you physically
24. You became homeless
25. You had a major financial problem
26. You got married
27. Your partner hurt your children physically
28. You attempted suicide
29. You were convicted of an offense
30. You were bleeding and thought you might miscarry
31. You started a new job
32. You had a test to see if your baby was abnormal
33. You had a result on a test that suggested your baby might not be normal
34. You were told that you were going to have twins
35. You heard that something that had happened might be harmful to the baby
36. You tried to have an abortion
37. You took an examination
38. Your partner was emotionally cruel to you
39. Your partner was emotionally cruel to your children
40. Your house or car was burgled
41. You had an accident
42. Is there anything else which is not on the list which has concerned you or required additional effort from you to cope since becoming pregnant?
